# Dynamic Parameters of Balance Which Correlate to Elderly Persons with a History of Falls

**DOI:** 10.1371/journal.pone.0070566

**Published:** 2013-08-05

**Authors:** Jesse W. Muir, Douglas P. Kiel, Marian Hannan, Jay Magaziner, Clinton T. Rubin

**Affiliations:** 1 Department of Biomedical Engineering, State University of New York, Stony Brook, New York, United States of America; 2 Institute for Aging Research, Hebrew SeniorLife, Harvard Medical School, Boston, Massachusetts, United States of America; 3 Epidemiology and Public Health, School of Medicine, University of Maryland, Baltimore, Maryland, United States of America; UCSD School of Medicine, United States of America

## Abstract

Poor balance in older persons contributes to a rise in fall risk and serious injury, yet no consensus has developed on which measures of postural sway can identify those at greatest risk of falling. Postural sway was measured in 161 elderly individuals (81.8y±7.4), 24 of which had at least one self-reported fall in the prior six months, and compared to sway measured in 37 young adults (34.9y±7.1). Center of pressure (COP) was measured during 4 minutes of quiet stance with eyes opened. In the elderly with fall history, all measures but one were worse than those taken from young adults (e.g., maximal COP velocity was 2.7× greater in fallers than young adults; p<0.05), while three measures of balance were significantly worse in fallers as compared to older persons with no recent fall history (COP Displacement, Short Term Diffusion Coefficient, and Critical Displacement). Variance of elderly subjects' COP measures from the young adult cohort were weighted to establish a balance score (“B-score”) algorithm designed to distinguish subjects with a fall history from those more sure on their feet. Relative to a young adult B-score of zero, elderly “non-fallers” had a B-score of 0.334, compared to 0.645 for those with a fall history (p<0.001). A weighted amalgam of postural sway elements may identify individuals at greatest risk of falling, allowing interventions to target those with greatest need of attention.

## Introduction

Incidence of falls escalates with age, events exacerbated by declines in muscle mass, strength, coordination and balance [1], [2]. When considered in concert with age-related decline in bone quality and quantity [3], this elevated risk of falling portends an increase in the incidence of injury, including fracture [4]. The increase in falls and concomitant injuries not only elevates rates of mortality, it poses significant economic and societal burdens to health care systems worldwide [5].

Poor postural control is recognized as a major contributor to fall risk, and individuals may rely upon a range of balance strategies to remain upright [6], [7]. Stable posture is maintained by a complex, integrated feedback from the visual, proprioceptive, and vestibular systems, as well as coordinated control by the neuromuscular system [8]–[10]. A commonly available – and relatively simple – method of assessing balance and instability is stabilogram analysis, which entails recording the ground reaction vector, known as center of pressure (COP) [11]–[13]. Measures of stabilogram magnitude, peak and average sway velocities, and root-mean-square (RMS) amplitude, are used as predictors of fall-risk [14], [15], while frequency domain characteristics have been proposed as an alternative way to express the results of COP measures to best capture postural control [16].

With age, diseases such as glaucoma, diabetic neuropathy, and sarcopenia, as well as age-related declines in hearing, diminish the quality of the integrated sensory input systems critical to stability. Inevitably, fall risk is ultimately a complex amalgam of many different system inputs. Nevertheless, regardless of cause, identifying specific components of balance that contribute to elevated risk may help to target interventional strategies or environmental modifications to reduce the occurrence of falls.

The principal objective of this work was to determine if there were differences in parameters of balance between a young and elderly cohort – both with and without a self-reported history of falls – and use these data to develop an algorithm based on these retrospective data to potentially – and isolate those specific components of the posture measurements which help identify individuals at increased risk of falls. We hypothesized that ‘elderly’ subjects would be less stable than younger subjects, and that those elderly subjects with a history of falls would have a COP signature distinct from age-matched non-fallers. The comparison of COP measures in elderly fallers vs. non-fallers, and their relationship to the young healthy cohort was then used to iteratively develop a fall predictor algorithm. It is hoped that data such as these may ultimately provide simple, relatively accessible diagnostic information from balance to prospectively identify those individuals at greatest risk of falls.

## Methods

### Study Sample

Study protocols and participation consents were reviewed and approved by the institutional human subjects review boards (IRBs) at each participating institution, including Hebrew SeniorLife (Harvard Medical School), National Aeronautics and Space Administration, University of Texas Medical Branch at Galveston, and Stony Brook University. After obtaining written informed consent from each volunteer in the trial, participants underwent a screening for eligibility including medical history, medication review, and a dual energy x-ray absorptiometry scan (*n.b.,* signed consent were collected for the “young” cohort by University of Texas Medical Branch and National Aeronautics and Space Administration, and for the “elderly” volunteers by Hebrew SeniorLife and Harvard Medical School). Those with osteoporosis (T-scores of <−2.5) were excluded from participation.

Postural control (balance) COP data were collected from 37 healthy young adults (23 males & 14 females), as well as 161 elderly subjects (52 males & 109 females) [17], [18]. The young cohort was recruited from the Houston area (part of a NASA-funded study on chronic bed rest), and the elderly cohort was recruited from the Boston area (part of an NIH-funded study on osteopenia in an aged population). As a principal goal of the NASA study was to determine if chronic bed rest potentiated poor balance, those with a self-reported history of falls were excluded from the study [15]. At baseline evaluation, elderly subjects that had fallen in the prior six-months were identified by questionnaire, in which a fall was defined as an event in which part of the body above the ankle touched the ground, including a fall on the stairs. Each member of the elderly group had to be at least 60 years of age, have a BMI below 30, have no prior history of pharmacologic treatment for osteoporosis, and have a dual-energy x-ray absorptiometry (DXA) based T-score (number of standard deviations above or below the “young” normal average) of between −1.0 and −2.5, regardless of fall history [17]. Elderly subjects were healthy, free-living, cognitively intact residents of independent living communities in the Boston area [19].

### Measurement of Postural Stability

To assess postural control, each subject was instructed to stand in a relaxed manner on a force plate (Kistler 9286AA, Winterthur, Switzerland), with feet at shoulder width, hands at their sides, and eyes open (closed-eye tests were not permitted by the IRB panels). It was requested of each person to hold this ‘relaxed stance’ for a period of four minutes. To provide a visual cue during the test, a 2 cm-diameter blue spot was placed on a white wall at eye level, 2 meters from the subject, while other visual interference such as lab equipment was removed from the line of sight to reduce distractions. During data collection a member of the research group stood behind the subject to intervene in case of lost balance or a fall during testing, though no falls occurred during testing. COP measures were recorded by the force plate with an eight-channel amplifier, an analog-digital converter, and Bioware 3.2.6.104 software. Data were over-sampled at 1000 Hz, and filtered with a second order Butterworth loss-pass filter at 50 Hz.

### Data Analysis

Data were analyzed using a custom MATLAB program (version 7.0.1, The MathWorks, Natick, Massachusetts) to calculate scalar parameters of COP magnitude and velocity, including maximal COP displacement from the centroid, root mean square (RMS) of COP displacement, maximal COP velocity, and RMS of COP velocity. Power spectral density calculations were performed to the total energy of the signal contained in the 0.2 to 1.0 Hz band which has been associated with the vestibular/somatosensory elements of postural control [20]. Finally, stabilogram diffusion analysis was performed to calculate the Short Term Diffusion Coefficient, a measure of the randomness of sway, the Long Term Scaling Exponent, a measure of the persistence of sway, and the critical displacement, a measure of the average displacement from the stabilogram's centroid which occurs before the postural control system switches from an open loop to a closed loop system [21].

To assess fall risk, z-scores for the elderly participants with a history of a fall and for those with no history of falls were calculated. COP measures of sway in the elderly participants were normalized using the equation:
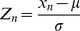



Z_n_  =  Normalized Z score for parameter *n*

x_n_  =  raw value to be standardized from the elderly group

μ  =  mean of the sample (calculated from young normal adults)

σ  =  standard deviation of the sample (calculated from young normal adults)

**Figure 1:**
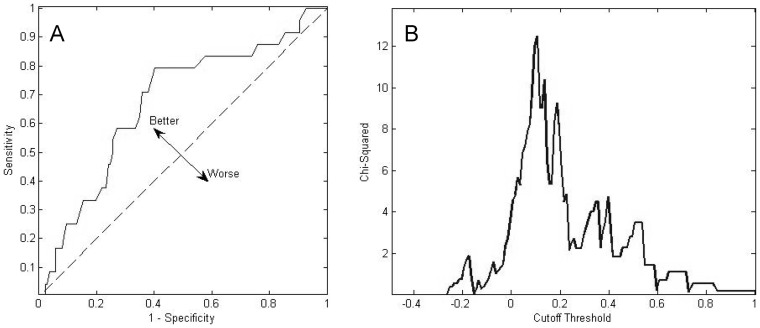
A: ROC plot of COP displacement variable. Sensitivity and 1-Specificity are plotted as the cutoff value is varied. The ideal location is in the upper left hand quadrant (1,0), where 100% of fallers are detected and 0% of non-fallers are identified (no false positives). The dotted line (line of no-discrimination) represents the location of a value with no predictive value (catches the same percentage of both fallers and non-fallers). B: Plot of chi squared value versus cutoff threshold. Values over 3.84 have a significance of p<0.05, and values over 10.83 have significance of p<0.001. Data from other variables result in similar values.

To calculate the “at risk” threshold value for each COP parameter, the normalized Z-scores of the elderly faller and non-faller groups were compared using receiver operating characteristic (ROC) analysis. The ROC analysis of the Z-score data to calculate cutoff thresholds (Fig. 1), and the value with the strongest specificity and sensitivity was then used as a cutoff, with any value above the threshold being labeled as “at risk.” The percentage of parameters tagged as “at risk” constituted the “B-score” of the subject, which was calculated by the following equations, with the B-score ranging from:

0; representing COP measures defined by the healthy young adult population, to

1; reflecting those at greatest risk of falls:
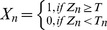



X_n_  =  “At Risk” label (1 if true, 0 if false)

T_n_  =  Threshold value for parameter n calculated from ROC analysis, and
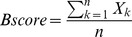



As the postural sway data in this study was non-parametric, the Kruskal-Wallis test was performed to compare multiple groups, and a Mann-Whitney test with post hoc comparisons adjusted for multiple testing using a Bonferroni correction to calculate between-pair significance. Statistical tests were performed using SPSS (version 14.0.0, SPSS Inc, Chicago, IL). As presented, all error bars indicate the standard deviation of the measure. P-values less than 0.05 were considered significant.

## Results

In order to use the information on prior falls in the prediction algorithm, elderly subjects were divided into two groups; those with a record of self-reported recent falls (n = 24; 14.9% of total elderly group) and those who had reported no falls in the prior six-month period (n = 137; 85.1% of total elderly group). Of the fallers, 9 were male (37.5%), while 43 of the non-fallers (31.3%) were male, indicating no tendency of males or females in this study sample to have a greater tendency to fall (p>0.05, χ^2^ = 0.35). However, there was not significant statistical power to determine the impact of gender on study outcomes.

When the “fallers” and “non-fallers” in the elderly cohort were considered in concert with the young healthy adults (n = 37), it yielded three groups of subjects for comparative analyses. The young adult group was significantly younger (34.9y±7.1) and taller (1.72m±0.09) than both elderly fallers (81.8y±7.4; 1.64m±0.14) and non-fallers (81.9y±6.6; 1.62m±0.10), with no significant difference in body weight (Table 1). No significant differences were found between fallers and non-fallers with respect to height, weight, or age. *Within* each of the groups, neither age, height, weight, nor gender was found to have any association with any specific postural control measure or history of recent falls. Thus, there are no body habitus or gender criteria which appear to predispose any given individual to falls.

**Table 1:** *Demographic Data* of the young adult, elderly without a history of falls, and the elderly population with a self-reported incidence of falls which had occurred in the prior six months.

	Young Adult	Elderly (No-Falls)	% Difference Young Adult	Elderly (Falls)	% Difference Young Adult
**N**	37	137		24	
**Gender**	24m, 13f*	43m, 94f		9m, 15f	
**Age (years)**	34.9±7.1*	81.9±6.6	134.7	81.8±7.4	134.4
**Height (meters)**	1.72±0.09*	1.62±0.10	−5.8	1.64±0.14	−4.6
**Weight (Kg)**	75.0±12.0	71.2±13.6	−5.1	71.6±12.3	−4.5

Percent differences relative to the young adult are also given.

There are no significant differences in age, weight or height between elderly non-fallers and those with a history of falls. While the young adult group was significantly taller than either of the elderly groups, there was no difference in weight between groups. * p<0.05, as compared to Elderly (No-Falls) and Elderly (Falls).

**Figure 2:**
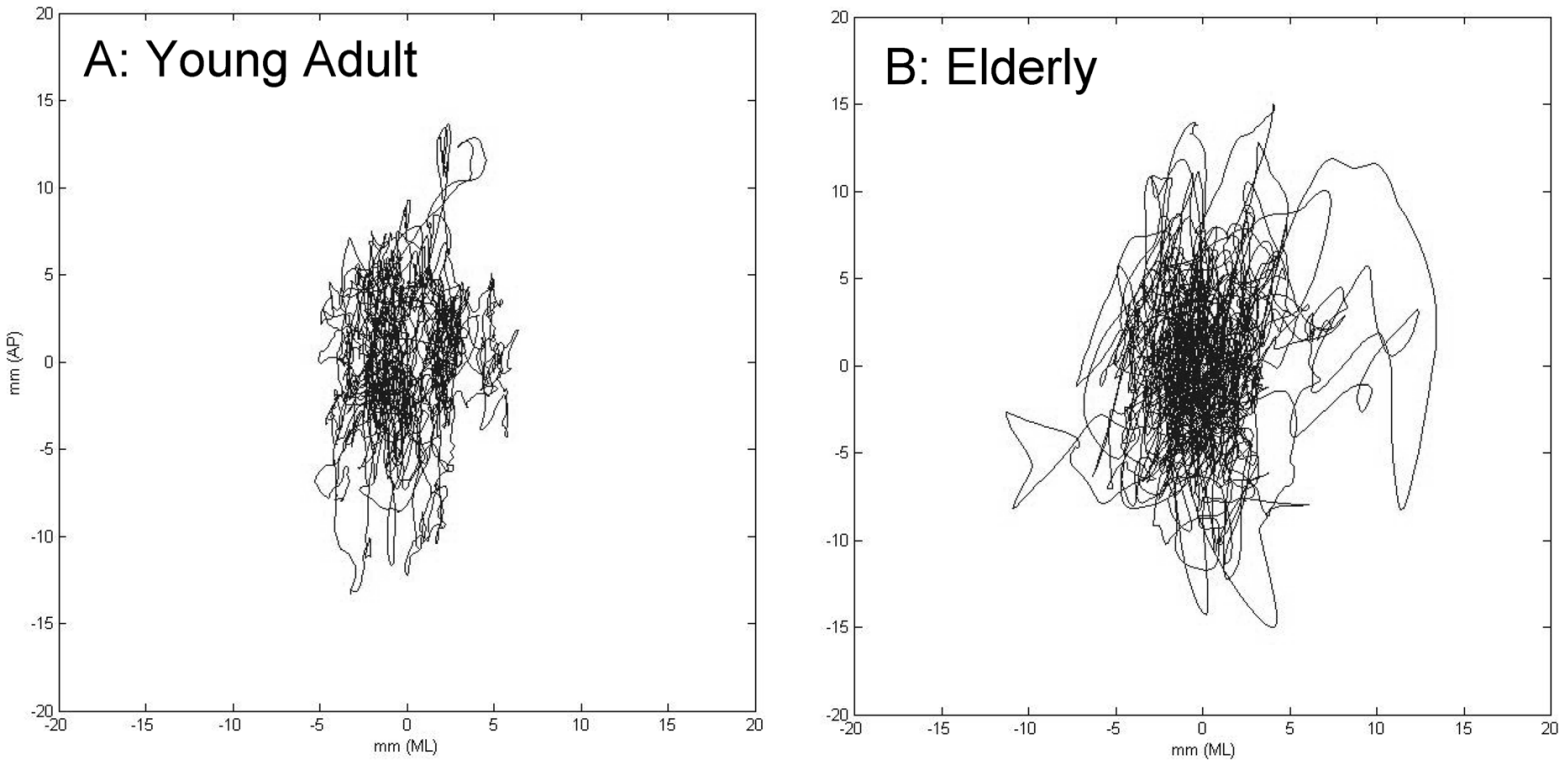
Stabilogram of a healthy young adult (A) and an elderly subject (B). The trace, collected over the four minute quiet-standing test period, represents movement of the center of pressure, in both the Medial-Lateral direction (side to side), and the Anterior-Posterior direction (front to back). Signs of poorer postural stability are evident in the aged subject, with a larger area of sway, as well as multiple excursions from center. As might be expected, with a longer path-length realized over the four-minute period in the elderly subject, peak sway velocities are also greater.

All participants completed the four-minute period of “quiet stance” on the force plate with no missing or out-of-range data. Stabilogram analysis revealed that, as compared to the young adults, several stability measures were significantly different in each of the elderly groups (Table 2; Fig. 2). While there were no significant differences between young and either elderly group in medio-lateral sway parameters, COP measures in the elderly revealed greater instability in multiple antero-posterior measures. For example, in the older group with recent falls, all measures except RMS displacement were significantly worse than in the young adults (e.g., maximal COP velocity was 2.7× greater in fallers than young adults; p<0.05). In comparisons between the two elderly groups, maximal displacement, diffusion coefficient, and scaling exponent were significantly worse in those with a history of fall (e.g., short term diffusion coefficient was 1.6 times greater in elderly fallers than elderly non-fallers).

**Table 2:** *Postural Stability Measures* of the young adult, elderly without a history of falls, and the elderly population with a self-reported incidence of falls which had occurred in the prior six months.

	Young Adult	Elderly (No-Falls)	% Change Young Adult	Elderly (Falls)	% Change Young Adult
**Maximal COP Displacement**	13.75±4.48^†*^	16.71±7.35^*#^	21.5	20.13±6.84^†#^	46.4
**RMS Displacement**	4.40±1.55	4.21±1.46	−4.3	4.77±1.53	8.4
**Maximal COP Velocity**	41.49±17.25^†*^	85.06±52.50^#^	105.0	112.35±68.46^#^	170.8
**Mean COP Velocity**	6.81±2.52 ^†*^	12.44±7.36^#^	82.7	16.26±9.64^#^	138.7
**RMS Velocity**	8.32±3.14 ^†*^	15.30±9.08^#^	83.9	20.17±11.84^#^	142.4
**Median Frequency**	0.86±0.16*	1.00±0.29^#^	16.3	1.08±0.45	25.6
**0 to 0.2 Hz Band Energy**	0.06±0.07^†*^	0.11±0.12^#^	83.3	0.14±0.14^#^	133.3
**0.2 to 1.0 Hz Band Energy**	0.34±0.27^†*^	0.71±0.81^#^	108.8	1.00±0.97^#^	194.1
**1.0 to 5.0 Hz Band Energy**	0.10±0.10^†*^	0.30±0.49^#^	200.0	0.54±0.95^#^	440.0
**Short Term Diffusion Coefficient**	5.41±3.83^†*^	13.23±14.33^*#^	144.5	21.68±18.17^†#^	300.7
**Long Term Scaling Exponent**	0.32±0.11^†*^	0.11±0.10^#^	65.6	0.07±0.08^#^	78.1
**Critical Displacement**	10.81±8.30^†*^	24.15±30.80^*#^	123.4	37.36±31.07^†#^	245.6

Percent changes relative to the young adult are also given. The majority of postural stability measures significantly deteriorated from young adult to the elderly non-faller group, and further eroded in the falls group. ^†^ p<0.05 from elderly (both groups); * p<0.05 from elderly fallers;^#^p<0.05 from young adult.

**Figure 3:**
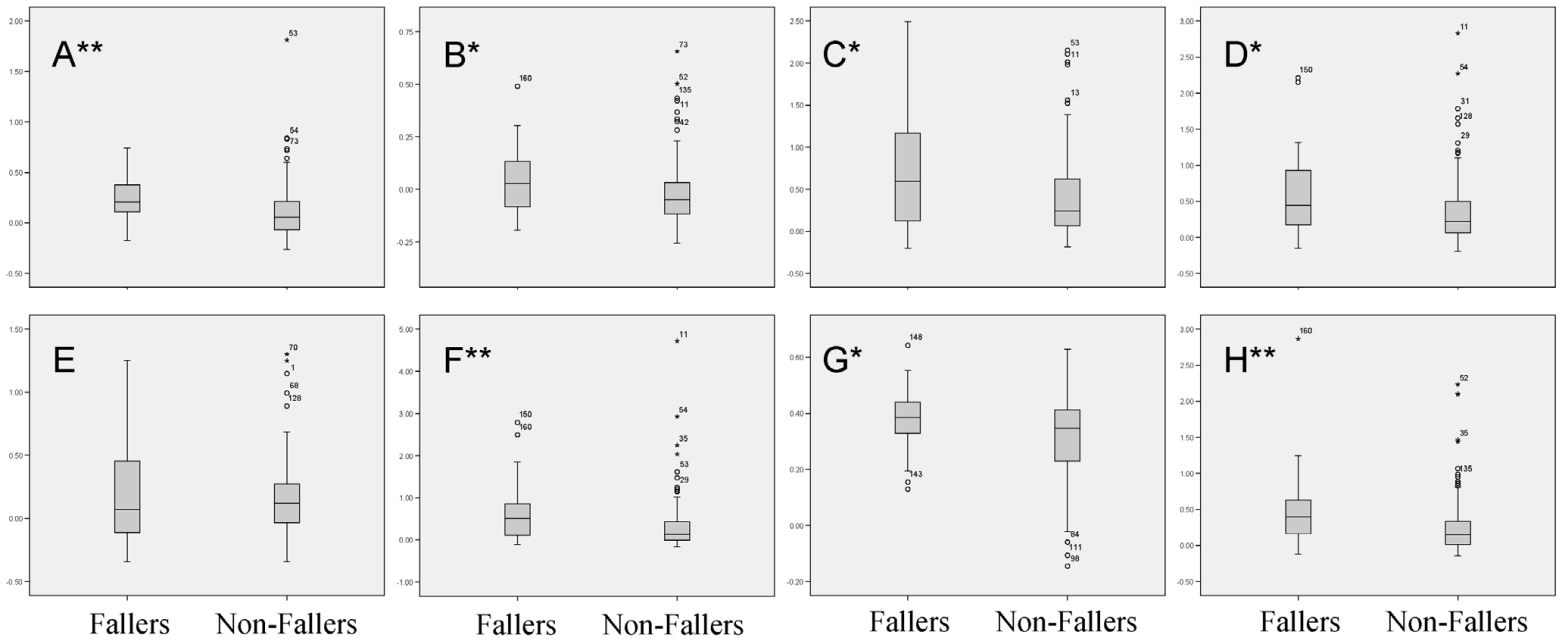
Box plots of Z-scores for maximal COP displacement (A), RMS of COP displacement (B), Maximal COP Velocity (C), RMS of COP Velocity (D), Median Frequency (E), Short Term Diffusion Coefficient (F), Long Term Scaling Exponent (G), and Critical Displacement (H). All measures but median frequency were significantly greater in fallers than non-fallers. The non-faller group had a number of outliers which were not excluded from data analysis. *p<0.05, **p<0.01.

**Figure 4:**
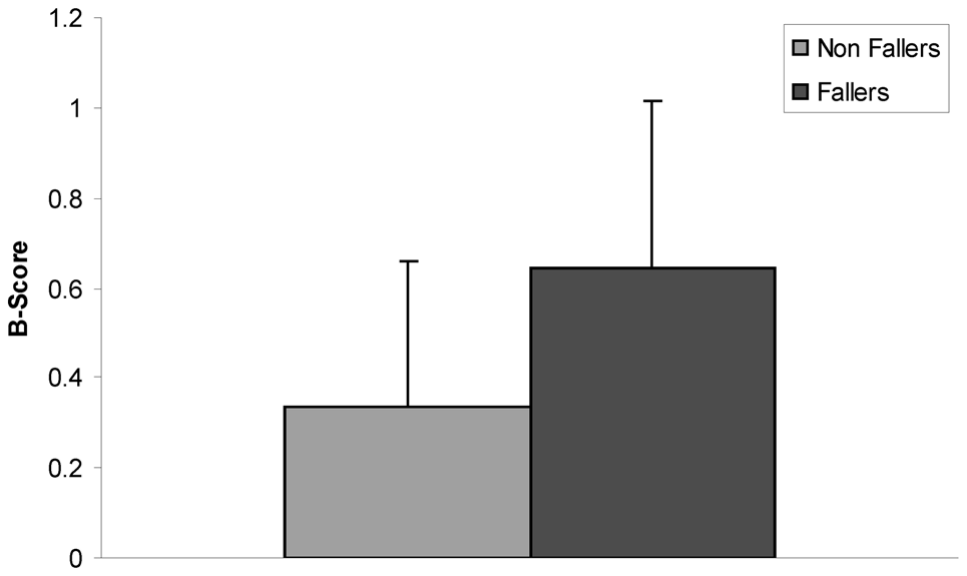
B-Scores of elderly subjects with and without self-reported falls. The B-score is based on an “idealized” postural sway profile, as established by a group of young healthy adults (B-score of zero). Those with at least one self-reported fall in the prior 6 months had a B-score of 0.645, compared to those without falls who had a B-score of 0.334 (p<0.0001).

As compared to young, healthy adults, the Z-scores of both elderly fallers and non-fallers were greater in measures of maximal and RMS displacement, maximal and RMS velocity, 0.2 to 1.0 Hz energy, short term diffusion coefficient, long term scaling exponent, and critical displacement (Table 3). With the exception of the short term diffusion coefficient, elderly with a history of falls had a Z-score approximately twice that of the non-fallers (Fig. 3). The B-score of elderly participants with a history of recent falls was found to be approximately two times greater than the B-score of elderly non-fallers (p<0.001; Fig. 4). ROC analysis of the B-score resulted in an idealized threshold value of 0.385, with a sensitivity of 70.8% and specificity of 73.7%.

**Table 3:** *Z-Scores for Elderly*, with and without a recent history of falls.

	Elderly (No-Falls)	Elderly (Falls)	% Difference
**Maximal COP Displacement**	0.11±0.27	0.23±0.25 *	109.1
**RMS Displacement**	−0.02±0.15	0.04±0.16 *	−300.0
**Maximal COP Velocity**	0.42±0.50	0.68±0.65 *	61.9
**Mean COP Velocity**	0.37±0.48	0.62±0.62 *	67.6
**RMS Velocity**	0.15±0.30	0.22±0.47	46.7
**Median Frequency**	0.11±0.28	0.19±0.33	72.7
**0 to 0.2 Hz Band Energy**	0.23±0.50	0.41±0.59 *	78.3
**0.2 to 1.0 Hz Band Energy**	0.32±0.81	0.71±1.58	121.9
**1.0 to 5.0 Hz Band Energy**	0.34±0.61	0.70±0.78 *	105.9
**Short Term Diffusion Coefficient**	0.32±0.15	0.38±0.12 *	18.8
**Long Term Scaling Exponent**	0.26±0.41	0.53±0.62 *	103.9

Percent difference between the groups are also given. The majority of the comparisons show significant differences. * p<0.05.

## Discussion

Falls in older persons are a major cause of debilitating injuries, fractures and even death; yet identifying those at greatest risk of falls – allowing a focus on individuals deserving of greatest attention – has proven difficult. The goal of this study was to use simple, readily accessible postural sway characteristics of a young, healthy adult sample with no history of falls to characterize “idealized” balance, and then use those parameters to identify similarities and differences of these COP measures to those from two groups of older individuals, those with a history of recent falls and those without. This information was then used to iteratively develop an algorithm, weighted by those parameters within the “falls” group that were most different from the young healthy adults, to differentiate those at higher risk of falls.

Many factors contribute to falls beyond postural sway, and include hazards as diverse as weather (e.g., ice) and obstacles in the home (e.g., rugs, electric cords, pets and stairs). As importantly, many limitations of the protocol must be noted. That the results are based on retrospective analysis of falls, and have not been validated by ‘predicting’ those that fall, is another limitation that must be emphasized. Extrapolating from ‘quiet stance’ to the dynamic conditions that are more closely associated with falls is yet another limitation that must be recognized, not fully offset by the goal of collecting ‘simple’ COP measures from achievable conditions such as standing, rather than more sophisticated challenges such as perturbations of stance, or measures made while walking on a variety of surfaces. But with the robust differences in balance measures identified between the young, the elderly non-fallers, and the elderly fallers, we also believe that an algorithm such as that presented here, from data collected under repeatable conditions, may ultimately help identify those at greatest risk, and thus identify those for whom environmental, physical or pharmacologic interventions might best be targeted.

Not all measures of balance derived from the COP stabilogram measures were able to distinguish between the young and elderly groups, and thus were not considered critical predictive indices of fall risk and were excluded in the B-score calculation. Additionally, some measures, such as medio-lateral sway magnitude and velocity, which were greater in the elderly group than in the young adult group (p<0.05), showed no difference between the elderly fallers and non-fallers, and were also omitted from the B-score calculation. Median power (p = 0.86) and the total energy of the COP signal contained in the 0 to 0.2 Hz (p = 0.07) and 1 to 5 Hz bands (p = 0.10), which have been associated with visual and proprioceptive/muscle control respectively [20], showed no significant difference in fallers and non-fallers, and were also omitted from the algorithm. It is certainly possible that a larger sample size may have revealed a significant difference in medio-lateral sway, and thus better justified its contribution to fall risk as identified by previous studies [22]. While power spectral density analysis identified differences between the young healthy adults and the elderly groups (both fallers and non-fallers) in median frequency and when compartmentalized into low, medium and high frequency ranges, the Z-score analysis could identify no differences between older participants with a history of falls and those with no falls, and thus was not included as an essential contributor in the B-score algorithm.

The use of ROC analysis of Z-score measures of sway allows for the prediction of fall risk as based on an iteratively defined threshold, such that subjects with a Z-score for a given parameter over the threshold would be tagged as being at greater risk for falls. However, analysis of the variance of sway parameters indicated that there was significant variation between subjects in terms of which sway parameters were above this threshold; one subject with a fall history may exceed the threshold for maximal displacement and velocity, while another may be over a threshold for RMS displacement, maximal velocity, and/or short term diffusion coefficient. This is most probably a reflection of the diverse range of factors which contribute to age-related declines in balance; an individual suffering from a compromise in vestibular feedback may exhibit different sway characteristics from a person suffering from mild neuropathy, while both may be at high fall risk [23]. Nevertheless, the greater the number of balance measures that were beyond a given threshold for a given parameter, the greater the likelihood that an individual was a member of the “falls” group. In fact, fallers exceeded the threshold in 5.17 out of 8 parameters, as compared to only 2.67 parameters in the non-faller group (p<0.001).

Considering the range of factors which distinguish ‘non-fallers’ within the elderly cohort from those with a self-reported history of falls, we worked towards incorporating a multitude of COP parameters into formulating the fall algorithm, rather than relying on any single component, to improve our accuracy in identifying those individuals at greatest risk of falls. Because of the nature of such a metric, it is certainly possible to iteratively change the weighting of any given parameter, or to add additional parameters to the calculation which were not measured in this study but may be related to fall risk, such as degree of neuropathy (monofilament score), visual acuity, or measures of mobility (sit-to-stand time). It would also be critical to consider parameters such as muscle strength and/or bone quantity and quality, to extend this falls-risk algorithm towards the formulation of a *fracture*-risk score, and could help account for those with low bone density who avoid fracture by avoiding falls vs. those with high bone density who are prone to fracture because of a tendency to fall.

The results of this study build upon the information gathered over the last two decades of fall-risk research. The ability to accurately predict fall risk with high specificity and sensitivity is still an ongoing topic, though the mechanisms of postural control have been well examined. Prospective studies showed a correlation between COP displacement and falls in the elderly, even in subjects without apparent balance issues [24]. And while quiet stance was examined here, it is important to emphasize that the use of perturbations have provided great insight into those at risk of falling, and future prospective trials which incorporate more sophisticated assessment of fall risk are certain to provide critical information on the reactive mechanics of stability and the effects of age-related degradation on individual balance strategies [25], [26].Another limitation of this study is the dependence of self-reporting of falls, the key parameter used to stratify the elderly groups into those with recent fall history or those with a limited history of falls. Omissions in reporting are inevitable due to lapses of memory, or a given subject’s reluctance to admit to a recent fall. Further, no consideration was given for the cause of fall (stumble, collapse, trip, slip, collision, etc.), nor was weighting of records enhanced for those with multiple falls over the six-month period. The elderly participants came from an ongoing trial of volunteers who fulfilled certain pre-specified inclusion and exclusion criteria [17], and thus may not be readily generalized to other samples of aging populations (e.g., frail elderly, nursing home cohorts, etc.). Further, using a relatively small group as the normal for Z-score analysis (37 young adults with no history of falls) is not necessarily optimal, as a larger group might improve confidence in the calculation of “idealized” balance parameters.

The limitations of focusing on a simple COP measure made from quiet “static” stance cannot be overemphasized, particularly considering the numerous other factors beyond control of balance, *per se,* that contribute to risk of falling, including dynamic parameters of locomotion. Indeed, there is a significant and growing body of work that uses sophisticated measures of gait, dynamic balance and strength, at the very least, as much higher-fidelity indices of fall risk [27]–[29]. It must be pointed out, however, that this study was designed specifically to approach COP as a relatively accessible and affordable assay and ideally enhance its ability to be used independently – or in conjunction with – more sophisticated measures of fall risk [23]. And certainly, it is entirely possible that those with a history of falls have already “modified” their balance control, and thus we are comparing stability measures from those with a “fear of falling” to those that have not yet fallen. Finally, it is important to emphasize that this algorithm has been iteratively “optimized” as driven by retrospective correlations to fall history, and has not been validated by prospectively predicting falls in the elderly subjects. Ultimately, validation of the B-score fall risk algorithm must be performed in a prospective study that can predict incident falls and can determine the accuracy of the prediction with both short and long-term follow-up [30]. Unfortunately, we are not able to address, here, the fidelity of self-reported falls, the nature of the falls that did occur, the differences that would arise in comparing fallers/non-fallers to more sophisticated measures of stability, or the inherent strategies that a young cohort may use to remain balanced as contrasted with an elderly population.

These findings reflect the diagnostic potential of weighting specific components of postural stability to more accurately identify those with a history of falls, and suggest that balance measures from healthy young adults may serve as an idealized “B-Score” referent to identify those at risk of falling, just as a bone density Z- or T-score may help identify those at greatest risk of fracture due to low quantity [17]. And just as a bone-density score is limited in identifying the source of low bone density (i.e., nutrition, genetics, activity-level, etc.), the B-score presented here is limited in that it does not identify the ‘source’ of deteriorating stability (i.e., neuromuscular, vestibular, visual, proprioception, etc). Recognizing the many limitations of this study, we do believe that efforts to prospectively validate a B-score based on some balance parameters may help to identify those at greatest risk of falling, and evaluate the efficacy of physical or pharmacologic interventions to directly diminish fall occurrence.

Certainly, postural control as measured during quiet stance must be quantified and combined with other risk factors for falls such as drug side effects, muscle weakness, visual acuity, temporary illness, locomotor instability and environmental factors (e.g., stairs, ice, rugs, pets). Future work could assess differential scaling of sway parameters, co-morbidity, and longitudinal analysis of additional falls, and determine how well fall risk correlates with fractures in the aged. Ultimately, by improving our ability to identify those at highest risk, it will be possible to focus targeted treatments and therapies, as well as prevention strategies and lifestyle modifications to reduce the incidence of injury causing falls.
